# Selective conversion of methane to cyclohexane and hydrogen via efficient hydrogen transfer catalyzed by GaN supported platinum clusters

**DOI:** 10.1038/s41598-022-21915-9

**Published:** 2022-11-01

**Authors:** Lida Tan, Hui Su, Jingtan Han, Mingxin Liu, Chao-Jun Li

**Affiliations:** 1 Department of Chemistry, and FQRNT Centre for Green Chemistry and Catalysis, McGill University, 801 Sherbrooke Street West, Montreal, QC H3A 0B8 Canada; 2 State Key Laboratory of Applied Organic Chemistry, College of Chemistry and Chemical Engineering, Lanzhou University, 222 Tianshui South Road, Lanzhou, 730000 Gansu China

**Keywords:** Chemistry, Energy science and technology

## Abstract

Non-oxidative liquefaction of methane at room temperature and ambient pressure has long been a scientific “holy grail” of chemical research. Herein, we exploit an unprecedented catalytic transformation of methane exclusively to cyclohexane and hydrogen evolution through effective surface-hydrogen-transfer (SHT) at the heterojunctions boundary consisting of electron-rich platinum cluster (Pt) loaded on methane-activating gallium nitride (GaN) host. The experimental analysis demonstrates that the interface-induced overall reaction starts with methane aromatization to benzene and surface-bound hydrogen initiated by the Ga–N pairs, followed by the hydrogenation of benzene to cyclohexane with surface-bound hydrogen. The in-situ activated hydrogen at electron-rich metal Pt cluster is crucial for the hydrogenation and enables an outstanding selectivity (up to 92%) and productivity (41 μmol g^−1^) towards cyclohexane and hydrogen evolution concurrently at 300 °C, which is well-delivered after 5 recycling runs.

## Introduction

**Figure 1:**
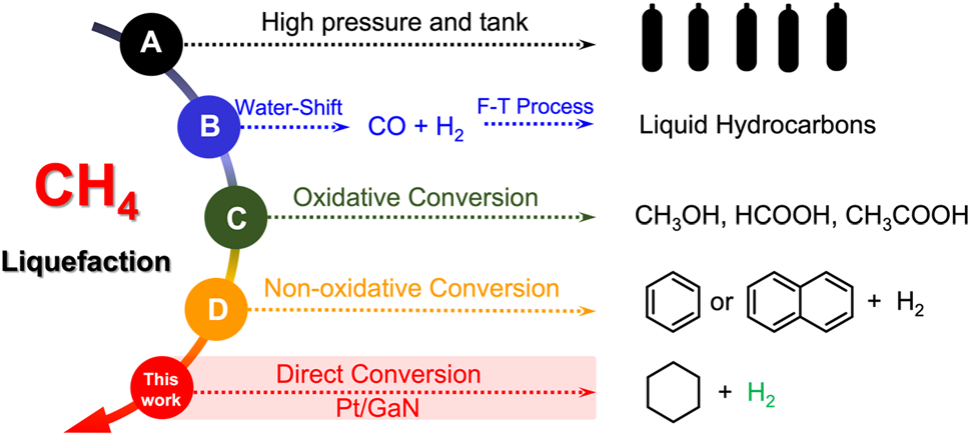
Methods for methane liquefaction. (**a**) High pressure tank for methane storage. (**b**) Typical two-steps process in large-scale industrial catalysis. (**c**) Oxidative conversion of methane into primary fuels and (**d**) non-oxidative conversion into aromatics. This work: a new methane liquefaction for cyclohexane synthesis over Pt/GaN catalyst via in situ surface-hydrogen-transfer (SHT).

The direct and selective liquefaction of methane, a vast natural carbon reserve and a major greenhouse gas, serving as a pivotal technology has been termed one of the “holy grails” in the scientific community^1,2^. The commonly available industrial liquefaction approach relies on high-pressure and/or low-temperature tank for ease of storage and transportation that requires harsh operating process and large energy input (Fig. 1a)^3,4^. To date, another successful application in industrial-scale methane liquefaction is to produce liquid hydrocarbons based on well-known two-step transformation involving the water–gas shift and the Fischer–Tropsch (F–T) processes (Fig. 1b)^5–7^. In view of economical cost, directly converting methane as building block feedstock into value-added liquid chemicals has received considerable attention for on-site transformation of methane of remote oil-rig^8–10^. In this context, tremendous effort has been devoted to exploit new reactivities of partial oxidation, oxidative condensation, and non-oxidative aromatization for the direct methane liquefaction into liquid fuels including methanol^11–13^, formic acid^14,15^, acetic acid^16–18^, even aromatics (Fig. 1c,d)^19–21^. However, unlocking novel and challenging transformation of methane to generate commodity chemicals is still rare and calls for new scientific routes.

On the other hand, cyclohexane as an important solvent and raw material has found its extensive applications in the plastic industry^22^, bulk chemicals synthesis^23^, and liquid organic hydrogen storage^24^, affording huge market potential. In particular, taking an example of Nylon production, the annual consumption of cyclohexane around the world is more than million-tons-scales^25^. Currently, the hydrogenation of benzene represents a mainstream methodology for cyclohexane production at the industrial-scale level^26,27^. Such a method is subjected to the over-dependence on the depleting petroleum resource, high environmental footprints, and elevated greenhouse gas emissions^28,29^. An efficient catalytic system has not yet been discovered to generate cyclohexane via one-step gas-to-liquid process from methane, a naturally abundant resource yet an environmentally concerning greenhouse gas. Therefore, rationally constructing and unlocking selective methane chemical liquefaction using advanced catalyst technology is of fundamental significance for the straightforward, economical, and sustainable production of cyclohexane.

The key challenge of converting methane into cyclohexane lies in the high dissociation energy of C–H bonds (104 kcal/mol) and the difficulty of preventing over-oxidation^30,31^. Our previous studies found that gallium nitride surface, even for commercial ones with rich gallium–nitrogen pair sites, is active for both strong C–H bond activation and subsequent aromatization to generate benzene in high yield as well as release detectable hydrogen gas^32,33^. To further obtain the desired cyclohexane, introduction of metal nanoparticles onto methane-active GaN support will, in principle, be powerful enough to trigger the subsequent benzene hydrogenation. Among all hydrogen-active metals, the supported Pt metal has been validated to afford strong hydrogenative capacity and even found its application in a challenging hydrogen storage-release process^34^. In the last decade, metal–semiconductor heterojunctions, particularly with well-defined interface engineering, can couple and even maximize catalytic functions of metal and the support concurrently through a joint boundary^35–37^. Such a conceptual “Two-in-One” protocol undoubtedly offers a facile toolbox for a vast array of various transformations that need multiple-function sites. For the present case, it is anticipated that rationally designed and fabricated metal–GaN interface in which methane-activation and hydrogenation areas are integrated simultaneously represents a promising solution for the challenging methane liquefaction process via cyclohexane.

Herein, we document an unprecedented and one-step methane conversion into cyclohexane and hydrogen gas based on robust platinum-metal clusters deposited on gallium nitride (Pt/GaN) for the first time. The utilization of the photochemical method successfully fabricates a bifunctional interface between well-dispersed Pt nanoclusters and GaN support^38^. This catalytic ensemble retaining a sufficient number of Ga–N pair sites is capable of activating inert methane molecules even after deposition of Pt nanoclusters. Meanwhile, the photoreduction deposited Pt nanoclusters with rich electron density possess excellent catalytic activity for the hydrogenation of benzene with the surface-bound hydrogen in-situ generated by the non-oxidative methane-aromatization, producing the target cyclohexane efficiently with up to 92% of selectivity and 41 μmol g^−1^ of primary product.

## Results

**Figure 2:**
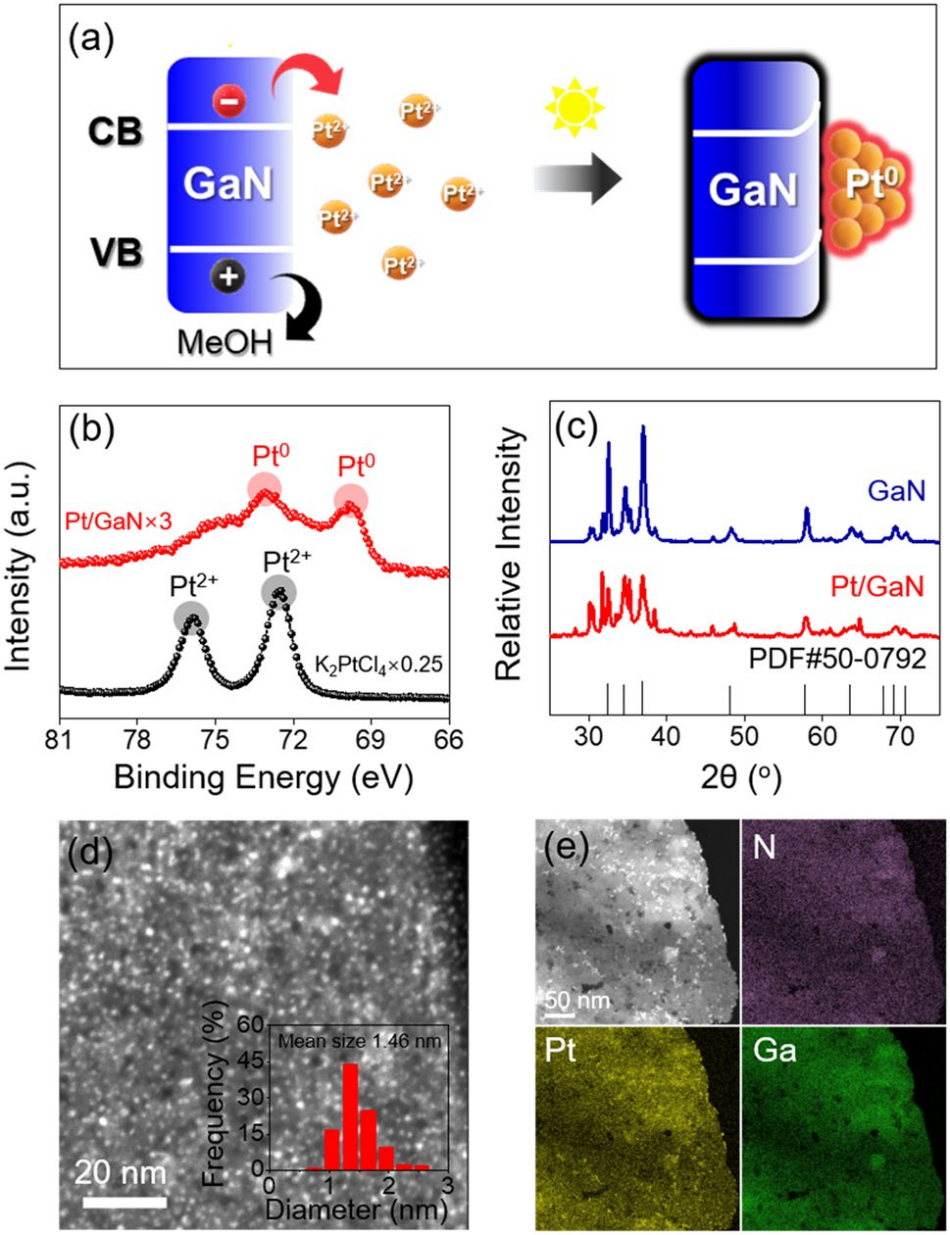
Construction and characterization of Pt nanoclusters coupled with GaN interface. (**a**) Photoreduction process of Pt/GaN catalyst. (**b**) Pt 4f XPS spectra for Pt/GaN and K_2_PtCl_4_ sample. (**c**) XRD patterns of Pt/GaN (red line) and commercial GaN (blue line). (**d**) Representative HAADF-STEM image of Pt nanoclusters size distribution and (**e**) EDS mapping files of Pt/GaN sample.

To obtain the highly active Pt/GaN interfacial catalyst, we chose a photochemical synthesis strategy in which methanol was used as photogenerated hole scavenger, enabling more photogenerated electrons to reduce the Pt precursor (K_2_PtCl_4_) to metallic Pt (Fig. 2a). The X-ray photoelectron spectroscopy (XPS) analysis indicated that Pt metal with zero-oxidation state was successfully introduced with assistance of GaN-semiconductor as photosensitizer (Fig. 2b and Fig. S1). As shown in Powder X-ray diffraction (XRD) results (Fig. 2c), commercial GaN powder with typical wurtzite structure was well maintained even after metal loading, demonstrating high tolerance and stability of GaN support against photochemical reaction media. At the same time, XRD pattern of Pt/GaN sample with no obvious characteristic peak for Pt nanoparticles reveals the presence of metal Pt possibly in the form of nanoclusters. The Brunauer–Emmett–Teller (BET) specific surface of Pt/GaN came out to be 12.31 m^2^/g whose value is similar with that of GaN (12.28 m^2^/g) (Fig. S2), accordingly excluding the Pt nanoparticles and aggregation in our samples. This deduction is further reflected by the high-angle annular dark-field scanning transition electron microscopy (HAADF-STEM) image of Pt/GaN in which ultra-small Pt nanoclusters with the average particle size of ~ 1.46 nm is indeed identified on the GaN surface (Fig. 2d). The energy-dispersive X-ray spectroscopy (EDS) mapping files show that Pt nanoclusters homogeneously dispersed on the entire GaN support (Fig. 2e), which is consistent with the HAADF-STEM results. These results demonstrate that the representative Pt/GaN sample is composed of ultra-small Pt cluster and typical wurtzite structured GaN. We further finely controlled the amount of Pt metal precursors in photoreduction reaction for optimizing the effective Pt/GaN boundary exposure to gas reactant. As-generated Pt_x_/GaN samples with different metal weight percentages (x = 0.5%, 1%, 2% and 4%) relative to GaN were used to catalyze the methane transformation.

**Figure 3:**
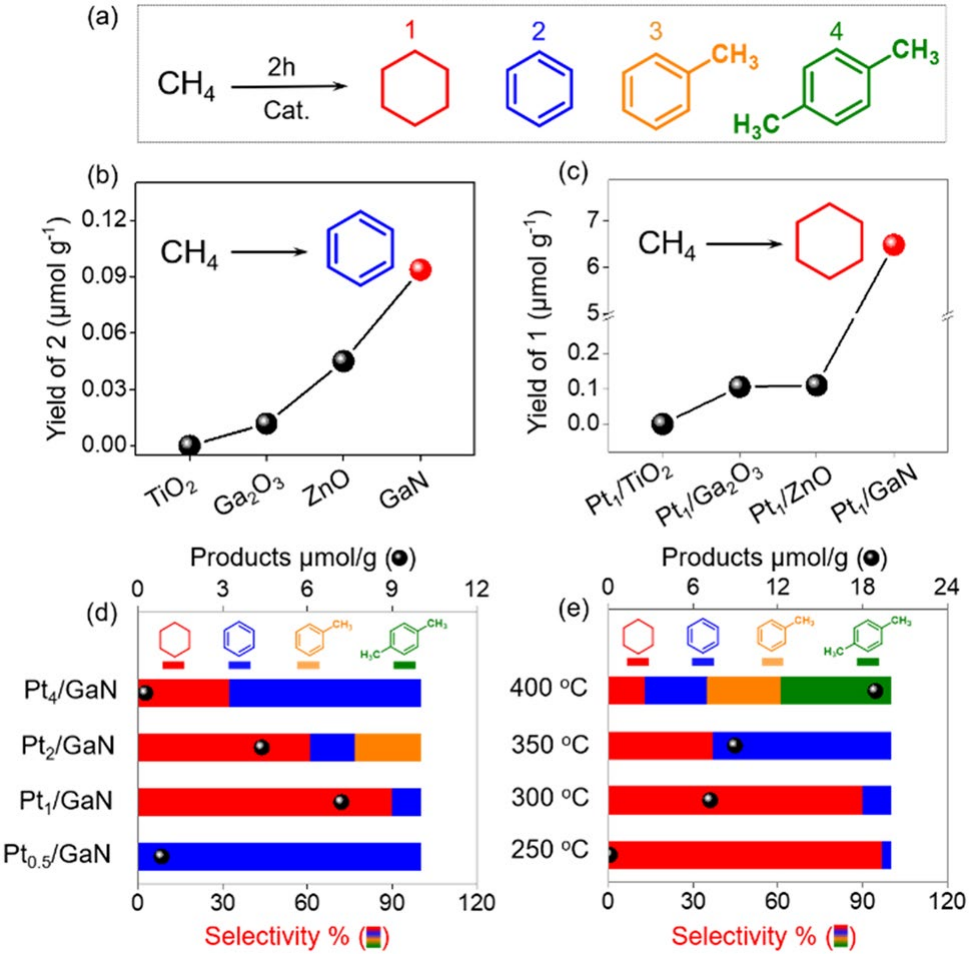
Methane liquefaction performance. (**a**) Schematic representation of methane-to-liquid products via thermal catalytic condition. (**b**) The yield of benzene and (**c**) cyclohexane over commonly used supports with and without loading Pt. (**d**) The selectivity and productivity of methane transformation by Pt_x_/GaN catalysts. (**e**) Catalytic performance of Pt/GaN under different reaction temperatures.

In our previous reported research^33^, commercial GaN powder can catalyze the non-oxidative aromatization conversion of methane to benzene generation in a detectable yield under thermal condition with the reaction temperature above 150 °C. Based on this experimental condition, we initially tested the thermal-catalytic methane non-oxidative conversion with different semiconductor-supports in a closed reactor at 300 °C for 2 h, in which 20 mg of Pt/GaN was homogeneously coated on the reactor wall using 2 mmol of high-purity methane as the single reactant. As shown in Fig. 3b (Entry 1–4 of Table S1), GaN support exhibited a higher yield of benzene compared to other supports for thermal methane conversion. In the final products analysis upon GC–MS and GC–TCD, only aromatics including benzene (blue) toluene (orange) and xylene (green) as side-products, (Fig. 3a) and hydrogen gas were generated (Fig. S3). Negligible carbon oxides and hydrocarbon gas but hydrogen gas was observed after the reaction even in the scale-up experiment (Figs. S3 and S4). These results demonstrated that GaN support indeed plays an essential role for activating inert methane molecules to form aromatic compounds that will be beneficial for the further cyclohexane and hydrogen gas generation.

Introducing Pt metal on to the methane-active semiconductor-support to form Pt/GaN interface can prompt the challenging generation of cyclohexane from methane with a high selectivity. No desired cyclohexane was formed at 300 °C by the methane-nonactive semiconductor supported Pt/TiO_2_ and Pt/C_3_N_4_ samples. (Fig. 3c and Entry 5 and 8 of Table S1). In contrast, Pt-modified methane-active supports showed noticeable production of cyclohexane under the same reaction conditions (Fig. 3c and Entry 6–9 of Table S1), in which Pt/GaN hybrids exhibits 25-fold higher catalytic performance of 6.49 μmol g^−1^ for methane conversion than those of well-controlled Pt/support (here are Ga_2_O_3_ and ZnO) heterojunctions. All the results suggest that GaN can serve as the ideal methane-active support to load Pt nanoclusters used for the hydrogenation of methane-aromatized benzene to cyclohexane.

The further optimization of metal loading on GaN surface was conducted to finely accommodate both electron density of Pt and electrostatic polarity of Ga–N pair for well matching yield rate of methane dehydrogenation and benzene hydrogenation, thus ensuring Pt/GaN with high chemoselectivity and productivity for methane-to-cyclohexane conversion (Fig. 3d). First of all, the exposure of sufficient Pt–GaN interfaces is a prerequisite to initiate the hydrogen-transfer process for cyclohexane synthesis from methane. The low metal content of Pt_0.5_/GaN sample mainly with enhanced methane-active Ga–N pairs (Fig. S1b) still could not generate any cyclohexane but more benzene than pure GaN sample (Entry 10 of Table S1). In combination with trace yield of PtO/GaN for cyclohexane synthesis, we can eliminate the positive contribution of Pt^2+^ species in Pt clusters for methane-to-cyclohexane transformation. The Pt concentration varying from 0.5 to 1 wt % is able to result in the generation of highly efficient Pt–GaN interface between electron-rich Pt clusters and strong Ga–N pair modified GaN support that has been confirmed by the downward shift of Pt XPS peaks and slight upshift of Ga XPS peaks from Pt_0.5_/GaN to Pt_1_/GaN (Fig. S1). Such “two-in-one” interface of Pt_1_/GaN coupled with hydrogenation-active Pt cluster and methane-active GaN therefore achieved the best-performance in both productivity (6.49 μmol/g) and selectivity (90%) toward cyclohexane as well as a considerable hydrogen gas production (5.5 μmol/g). Such an excellent reactivity far outperforms all reported catalytic systems even handled under above 600 °C (Table S5). Further increasing the Pt loading on the surface of GaN inevitably induced aggregation of metal clusters that can not only impede photogenerated electron transfer from GaN to Pt (Fig. S1) but also block catalytic interface, thus losing their catalytic reactivity for Pt_2_/GaN and Pt_4_/GaN (Fig. 3d and Entry 11–12 of Table S1). These results demonstrate the synergetic role of highly dispersed Pt_1_/GaN interface with strong Ga–N pair modified GaN support and electron-rich Pt clusters on simultaneously catalyzing the methane aromatization and the subsequent hydrogenation for synthesizing cyclohexane in an ultra-selective manner.

The reaction temperature has imperative impact on delicately balancing methane aromatization and benzene hydrogenation rates with the aim of converting more methane to cyclohexane via the proposed methane-benzene-cyclohexane process (Fig. 3e and Entry 14–16 of Table S1). Decent cyclohexane selectivity (97%) but tiny cyclohexane productivity was observed at a lower temperature of 250 °C (Entry 14 of Table S1). Further increasing reaction temperature for breaking thermodynamic limitation at low temperature benefits to enhance methane conversion applied in practical manufacture. When the reaction temperature was raised to 300 °C (Entry 9 of Table S1), the cyclohexane generation rate reaches an optimal value (6.49 μmol g^−1^) in the temperature-dependent reactivity profiles wherein superior chemoselectivity is comparable to the one at 250 °C. But higher temperatures from 300 to 350 °C even 400 °C were found to lower selectivity (42% and 12%) as well as corresponding yield (3.32 μmol g^−1^ and 2.39 μmol g^−1^) toward cyclohexane in our catalytic case (Entry 15–16 of Table S1). Consequently, following experiments are carried out under 300 °C. Such reaction temperatures-dependent trade-off correlation between cyclohexane selectivity and productivity presented by the optimized Pt/GaN again implies that synergistic mechanism working at the Pt/GaN interface is likely responsible for this novel methane-to-cyclohexane process.

At last but not the least, well-designed Pt_1_/GaN catalyst is still capable of maintaining good selectivity (93% and 92%) and displaying higher productivity (11 μmol g^−1^ and 41 μmol g^−1^) after updating batch reactor from 50 to 100 mL even 500 mL (Entry 17–18 of Table S1). Those induce that the utilization of Pt_1_/GaN catalyst in combination with effective reactor potentially enable scale-up production of cyclohexane in an appreciable yield even used for practical application.

**Figure 4:**
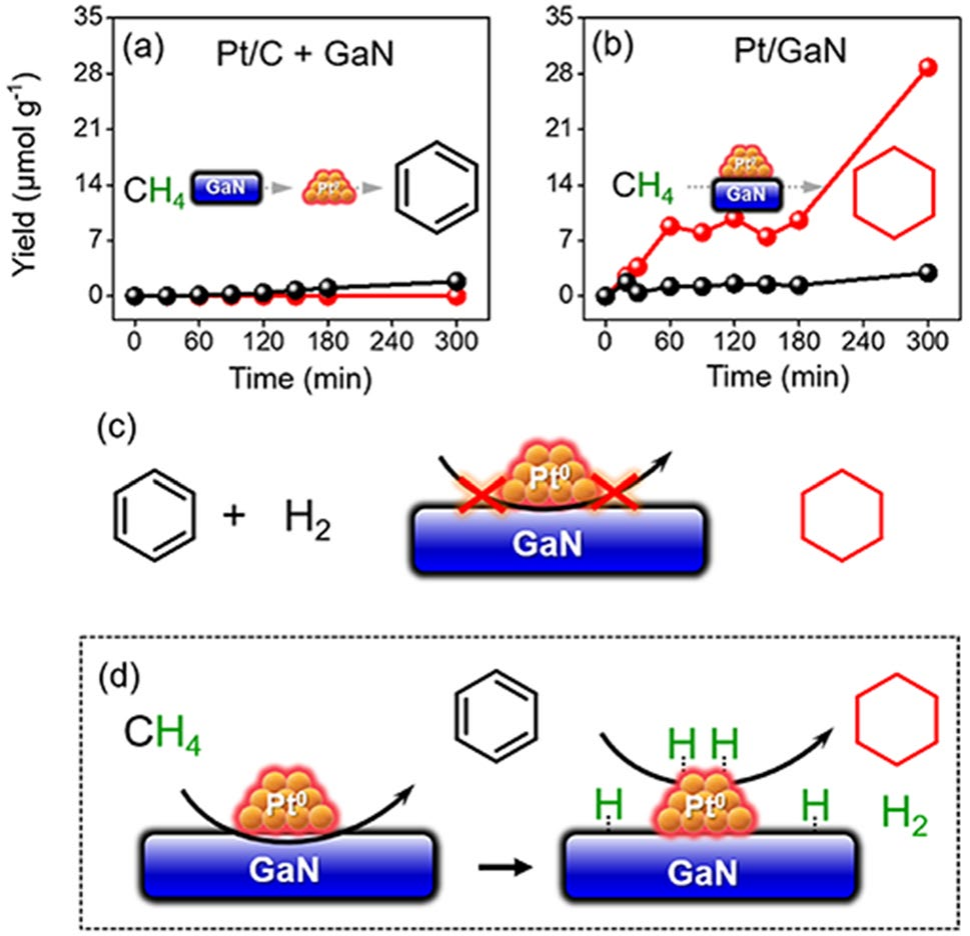
Methane conversion mechanism studies. (**a**) Time-dependent yield of cyclohexane (red sphere) and benzene (black) with the mixture of commercial Pt/C and GaN and (**b**) Pt/GaN and as catalysts. (**c**) Yield of cyclohexane over Pt/GaN via benzene reacting with hydrogen gas and the corresponding schematic illustration. (**d**) Proposed methane conversion pathway for production of cyclohexane catalyzed by Pt/GaN interface.

To gain in-depth insight of the catalytic methane transformation process on Pt/GaN surface, we compared time-dependent reaction performance for as-formed Pt/GaN and a mechanical mixture of commercial Pt/C and bare GaN (Fig. 4a,b and Tables S2, S3) of which there is similar Pt loading. We found that only trace non-oxidative methane conversions occurred in the presence of Pt/C and GaN mixture even after 5 h under the standard thermal conditions owing to non-existence of the effectively integrated interface between Pt/C and GaN (Fig. 4a). In contrast, the yield of cyclohexane has obviously increased after using Pt/GaN and was accompanied with a small amount of benzene generated from aromatization of methane (Fig. 4b). Such results demonstrated that the overall reaction pathway indeed involves successive aromatization and hydrogen transfer steps near the boundary of Pt clusters and GaN support. Further control experiment was carried out to verify the essential feature of such a surface hydrogen auto-transfer process where the equivalent amounts of benzene and H_2_ gas are as much as those of theoretical production of our standard methane liquefaction reaction over Pt/GaN (Fig. 4c). As we expected, the hydrogenation of benzene towards cyclohexane with hydrogen gas did not take place significantly (Fig. S8). These results unravel that the in-situ generated H-atoms on the surface of GaN via methane dehydroaromatization are quite prone to migrate to the adjacent Pt metal surface for the subsequent hydrogenation driven by active metal hydride intermediate rather than undergoing two separate steps of dehydrogenation–hydrogenation to accomplish the successive hydrogenation of benzene (Fig. 4d). Consequently, a hypothetic hydrogen-transfer mechanism here is presented for direct methane conversion to cyclohexane. The direct activation C–H bond of methane could happen through Ga–N pair promoted C^δ–^H^δ+^ interaction model^33^ for the formation of surface-bond CH_x_ radical and hydrogen radical intermediates. Then dimerization and deep cyclization reaction would proceed, producing benzene on the Pt/GaN interface, which could be further hydrogenated by adjacent Pt–H into final cyclohexane.

**Figure 5:**
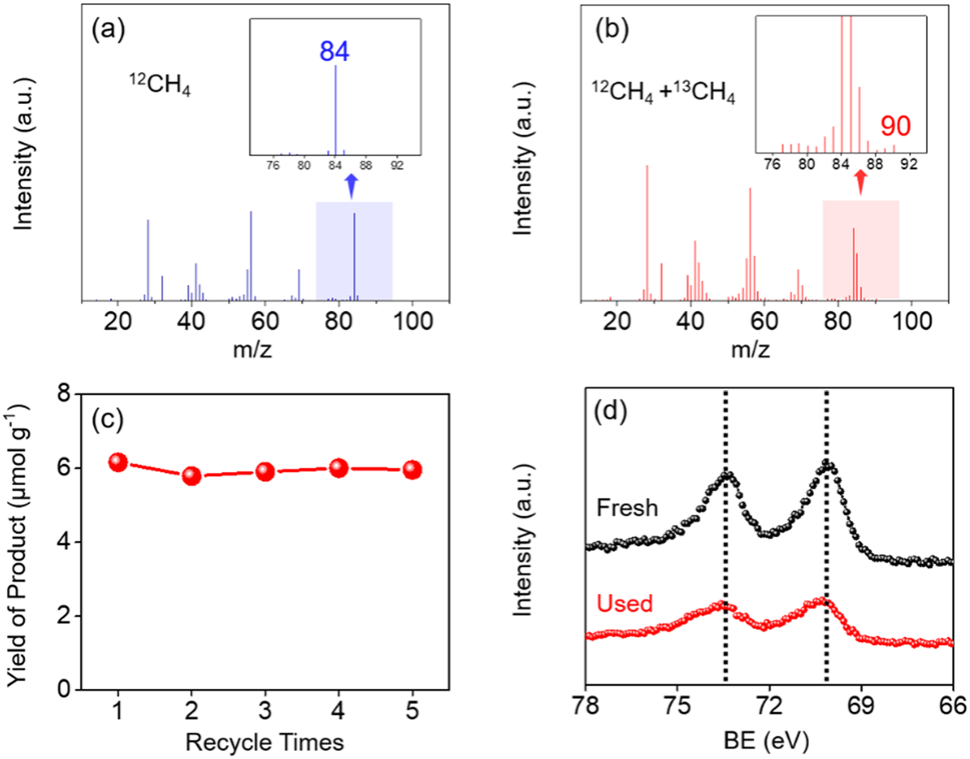
Reusability of Pt/GaN for catalyzing methane-to-cyclohexane. (**a**) GC–MS spectra of cyclohexane derived from ^12^CH_4_ and (**b**) the mixture of ^12^CH_4_ and ^13^CH_4_. (**c**) Yield of cyclohexane over reused Pt/GaN. Reaction conditions: 2 mmol of methane, 20 mg of catalyst, 300 °C, 2 h. (**d**) Pt 4f XPS spectra of fresh and used Pt/GaN.

Given the nature of heterogeneous catalysts, the Pt/GaN interface showed good recyclability without obvious attenuation in terms of production rate of cyclohexane under the thermal condition for up to 5 cycles (Fig. 5c and Entry 1–5 of Table S4). In present catalytic system, no obvious carbon allotrope deposition on the surface of catalyst was identified (Figs. S6, S7) before and after reaction. Performing control and isotopic experiments offer direct evidence for the carbon resource of cyclohexane indeed produced by methane conversion rather than other carbon contamination. It is noted that no detectable cyclohexane product was found under a standard condition just by switching methane with argon. More importantly, the comparison results obtained in ^12^CH_4_ and ^13^CH_4_ isotopic labeling conversion (Fig. 5a,b and Fig. S9) undoubtedly demonstrates the existence of ^13^C labeling cyclohexane from direct methane conversion catalyzed by Pt/GaN. Simultaneously, the morphology, crystal structure, and electron density states of fresh and the used catalysts stay the same as reflected by the results of SEM, TEM, XRD and XPS (Fig. 5d and Figs. S10–S12) All of those suggest the considerable stability of highly active Pt/GaN boundary.

In summary, we have uncovered a novel means for methane liquification via the highly selective generation of cyclohexane, by using Pt clusters supported on GaN as catalysts. The method converts methane gas into liquid cyclohexane and hydrogen with extremely high selectivity (up to 92%) and productivity (41 μmol g^−1^). Homogeneously dispersed Pt clusters on GaN are essential to achieve methane activation and the subsequent benzene hydrogenation via surface-hydrogen-transfer (SHT) process with the surface bound H-atoms for efficient production of cyclohexane. The catalyst system is very stable and can be reused for multiple times without diminishing the catalytic activity. As the catalyst was immobilized on the flask-wall in the present study, the method can be adapted readily into flow systems with existing facility for large scale applications. Furthermore, considering the state-of-art industrial hydrogen production is mostly from methane via the highly energy-intensive “steam-reforming”^39^, the current method allows the concurrent generation of hydrogen gas as a side product while producing high-valued cyclohexane. Such a green and sustainable conversion of low-pressure methane to value-added and portable liquid products with high selectivity exhibits great application potentials in large-scale industrial practice, as well as provides a potential means to capture this major greenhouse gas.

## Methods

### Materials

Commercial GaN catalysts (99.9% purity) were purchased from Sigma-Aldrich and used without further treatment. Methane (99.99% purity) was purchased from Air Liquide. All metal precursor (K_2_PtCl_4_) and other catalyst supports (Ga_2_O_3_, TiO_2_, Al_2_O_3_, C_3_N_4_) are commercially available compounds, which were purchased from Sigma-Aldrich and used without further purification.

### Synthesis of Pt_x_/GaN

Pt/GaN was prepared based on the photodeposition method as reported^1^. In the typical synthesis of Pt_x_/GaN (x = 0.5%, 1%, 2% and 4%), K_2_PtCl_4_ (0.50 mg, 1.00 mg, 2.00 mg, and 4.00 mg) was dissolved in deionized water (6.00 mL) and methanol (2.00 mL) as stock solution. 20 mg of the GaN nanoparticles was dispersed in 4 mL of the stock solution in a quartz tube and was stirred under photoirradiation of xenon lamp (PE300 BUV) for 3 h in argon gas. The suspension was collected by centrifugation, and was washed with distilled water and methanol for three times. The final sample was obatined after drying under vacuum at 300 °C for 1 h and 100 °C overnight.

### Synthesis of Pt/Ga_2_O_3_, Pt/TiO_2_, Pt/Al_2_O_3_, and Pt/C_3_N_4_

The depositions of Pt on other catalyst supports (Ga_2_O_3_, TiO_2_, Al_2_O_3_, and C_3_N_4_) were based on the photodeposition method as reported^1^. During the preparation, 1.00 mg K_2_PtCl_4_ was dissolved in deionized water (6.00 mL) and methanol (2.00 mL) as stock solution. 20 mg of the catalyst supports was dispersed in 4 mL of the stock solution in a quartz tube and was stirred under photoirradiation for 3 h under a xenon lamp (PE300 BUV) in argon environment. The suspension was collected by centrifuge, washed with distilled water and methanol several times, and dried under vacuum at 300 °C for 1 h and 100 °C overnight.

### Catalyst activity test of methane liquification reaction

All the tests were performed using Schlenk glassware or vacuum line techniques. Methane in this experiment was dried by passing through the column of MgSO_4_ and CuSO_4_ before the catalyst activity test. The catalyst activity test was performed in the 50 mL round bottom reactor at 250 °C, 300 °C, 350 °C and 400 °C, respectively. 20 mg of the catalysts was dispersed evenly at the bottom of the reactor, and the closed reactor was heated to 400 °C under vacuum for 2 h to remove the remaining water and other gas molecules (O_2_) on the surface of materials. The reactor was cooled to room temperature under vacuum. The catalyst was purged with methane and was vacuumed for three times before 2 mmol of methane was injected. During the reaction, the reactor was heated to the desired temperature and kept for 2 h. The organic products were analyzed and quantified by gas chromatography-mass spectrometry (GC–MS). For the reusability test, the used catalysts were evacuated at 500 °C under vacuum for 3 h after each run to remove all the remaining reactant and products. After that, the reactor was cooled to room temperature under vacuum. The catalysts were purged with methane and were vacuumed for three times before 2 mmol of methane was injected.

### Catalyst characterization

The bright field transmission electron microscopy (TEM) observations were carried out on FEI Tecnai G2 F20 S/TEM at accelerating voltage of 200 kV. The high-angle annular dark-field scanning transmission electron microscopy (HAADF-STEM) characterization was carried out on a Hitachi HD2700 Cs-corrected STEM, which was used with a cold field emitter operated at 200 kV and with an electron beam diameter of ~ 0.1 nm. Spectroscopic mapping by energy dispersive X-ray (EDX) spectroscopy was performed using a 60 mm^2^ silicon drift detector from Bruker. The scanning electron microscopy (SEM) was carried out on a FEI Quanta 450 Environment scanning electron microscopy (FE-ESEM) with EDAX Octane Super 60 mm^2^ SDD and TEAM EDS Analysis system. The X-ray photoelectron spectroscopy (XPS) was conducted on an ESCALAB 250 X-ray photoelectron spectrometer with a monochromated X-ray source (Al Kα hv = 1486.6 eV), and the energy calibration of the spectrometer was performed using C 1 s peak at 284.8 eV. The powder X-ray diffraction (XRD) patterns were obtained on a Bruker DD8 Advanced diffractometer with Cu Kα radiation (*λ* = 1.5418 Å). Raman spectrum was performed on Confocal Raman Microscope (Alpha300, Witec, Ulm, Germany) with a piezo scanner (P-500, Physik Instrumente, Karlsruhe, Germany). The scattered light was measured via a thermoelectrically cooled CCD detector (DU401A-BV, Andor, Belfast, North Ireland) behind the spectrometer (UHTS 300, WITec, Ulm, Germany).

### Calculation of selectivity

The selectivity of cyclohexane is determined as the ratio of moles of cyclohexane produced in the reaction to the total mole of all hydrocarbon products in terms of carbon (1).

1 $${{\text{Selectivity of cyclohexane }}}=\frac{{{\text{Moles of cyclohexane produced in terms of carbon}}}}{{{\text{Moles of all hydrocarbon products in terms of carbon}}}} \times 100 \%$$

## Supplementary Information

Supplementary Information.

## Data Availability

All data generated or analyzed during this study are included in this published article and its supplementary information files.
