# Supplementary material for: Selective conversion of methane to cyclohexane and hydrogen via efficient hydrogen transfer catalyzed by GaN supported platinum clusters
Source: Sci Rep. 2022 Nov 1;12:18414. doi: 10.1038/s41598-022-21915-9 (PMC9626580; doi:10.1038/s41598-022-21915-9)
Supplement: Supplementary file 1 — Supplementary Information. [file 41598_2022_21915_MOESM1_ESM.pdf]

Supplementary Information

Selective conversion of methane to cyclohexane and hydrogen via efficient hydrogen transfer catalyzed by GaN supported platinum clusters

Lida Tan, [a, ‡] Hui Su, [a, ‡] Jingtan Han, [a, ‡] Mingxin Liu,[a, b, ‡] Chao-Jun Li, [a, *]

[a] Department of Chemistry, and FQRNT Centre for Green Chemistry and Catalysis, McGill University, 801 Sherbrooke Street West, Montreal, QC H3A 0B8, Canada.

[b] State Key Laboratory of Applied Organic Chemistry, College of Chemistry and Chemical Engineering, Lanzhou University, 222 Tianshui South Road, Lanzhou, Gansu, China 730000.

‡ These authors contributed equally to this work

* E-mail address: cj.li@mcgill.ca

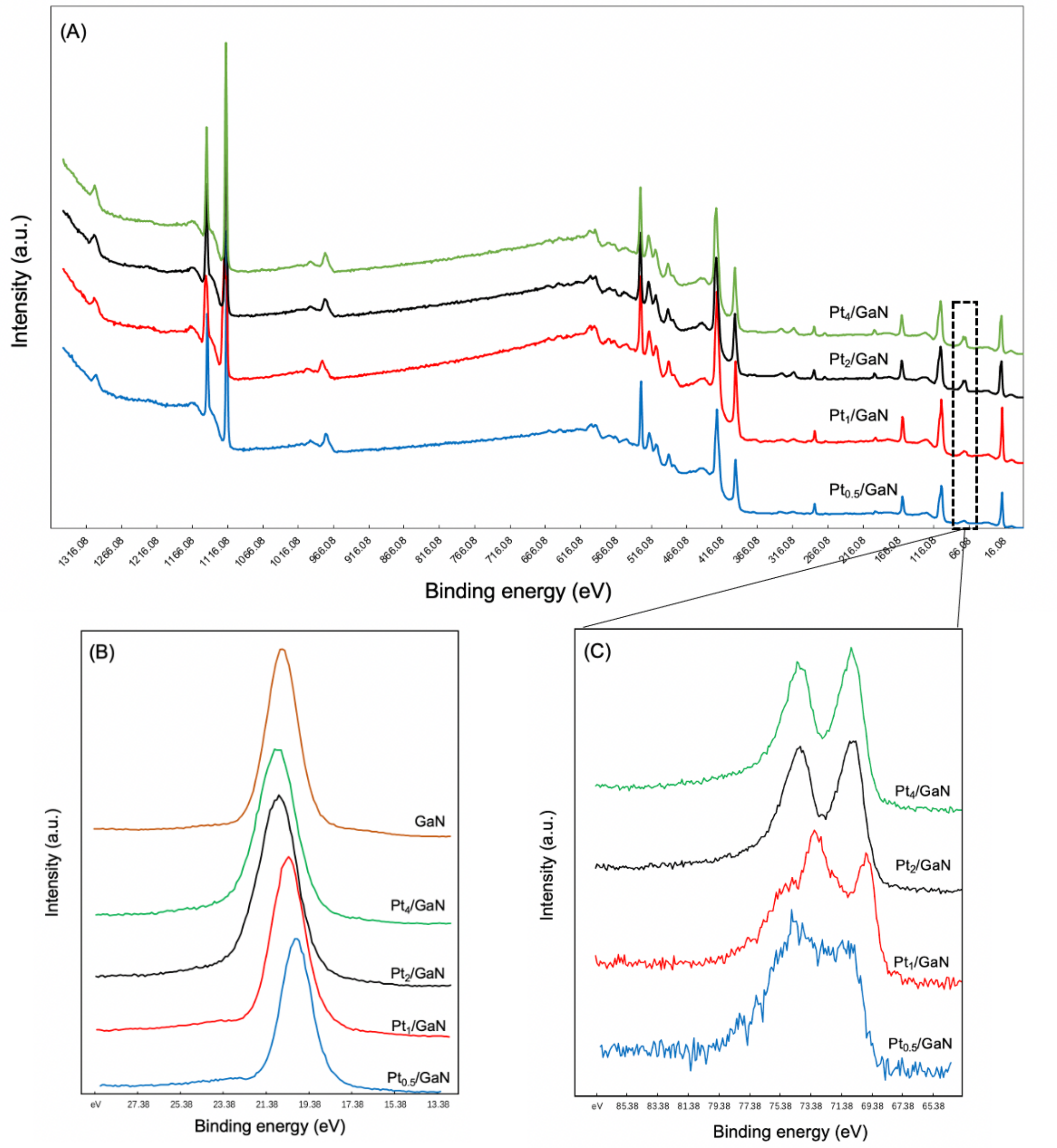

Figure S1. Representative XPS spectroscopy of $Pt_{0.5}$/GaN, Pt_1/GaN, Pt_2/GaN, and Pt_4/GaN samples. (**A**) Full XPS spectra, (**B**) Ga 3d XPS spectra, (**C**) Pt 4f XPS spectra.

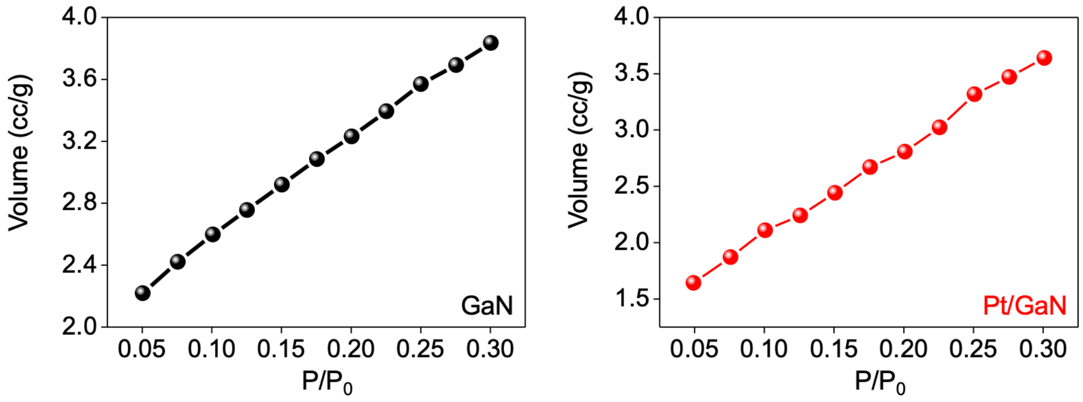

Figure S2. The plot of Brunauer-Emmett-Teller (BET) surface area measurement of GaN and Pt/GaN.

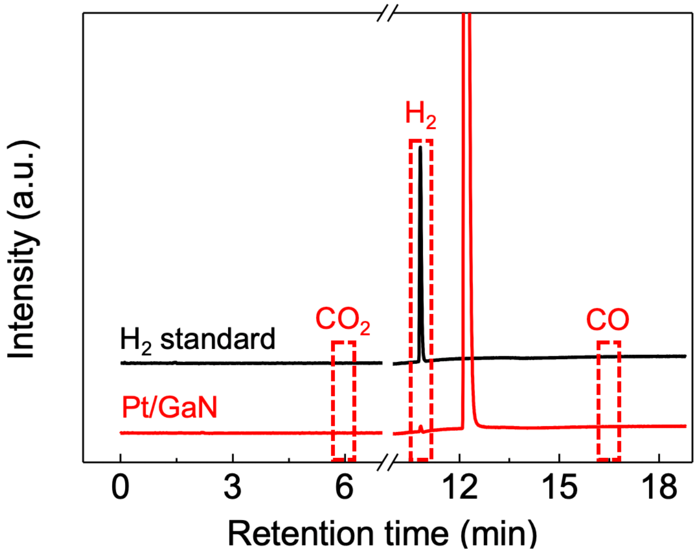

Figure S3. Representative GC-TCD chromatography of the gas sample obtained by Pt_1/GaN at 300 °C after 2 h showed that the main by-product is H_2 without generation of oxidation and/or over-oxidation products like carbon oxide and/or carbon dioxide, suggesting that GaN along with strong ability for direct activation of methane can push methane aromatization conversion to generate benzene and H_2 gas.

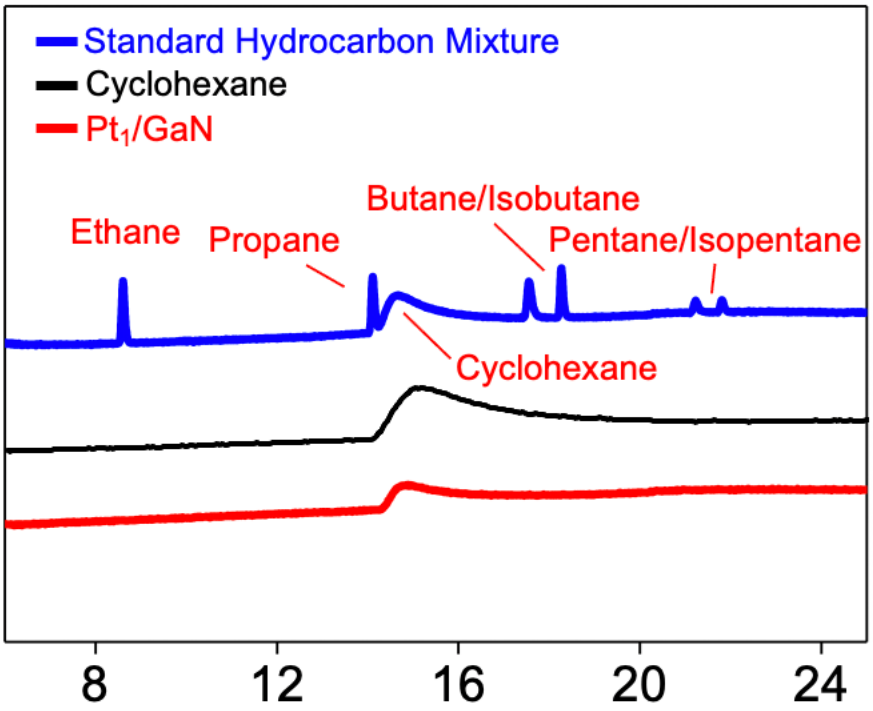

Figure S4. Representative GC-TCD chromatography of cyclohexane, standard hydrocarbon mixture (C1-C6) and the gas sample obtained by Pt_1/GaN under 500 mL batch reactor.

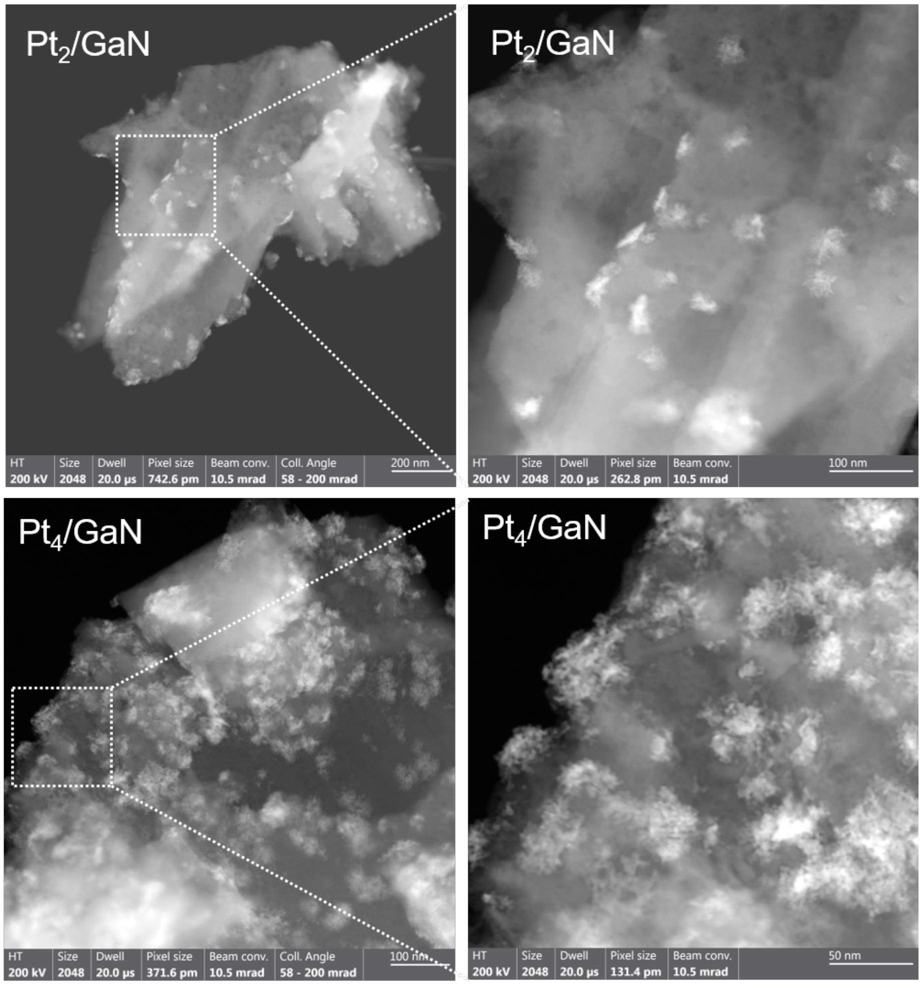

Figure S5. Typical HAADF-STEM images of Pt_2/GaN and Pt_4/GaN samples. It is observed that high metal loading samples show obvious metal aggregation to form metal "island".

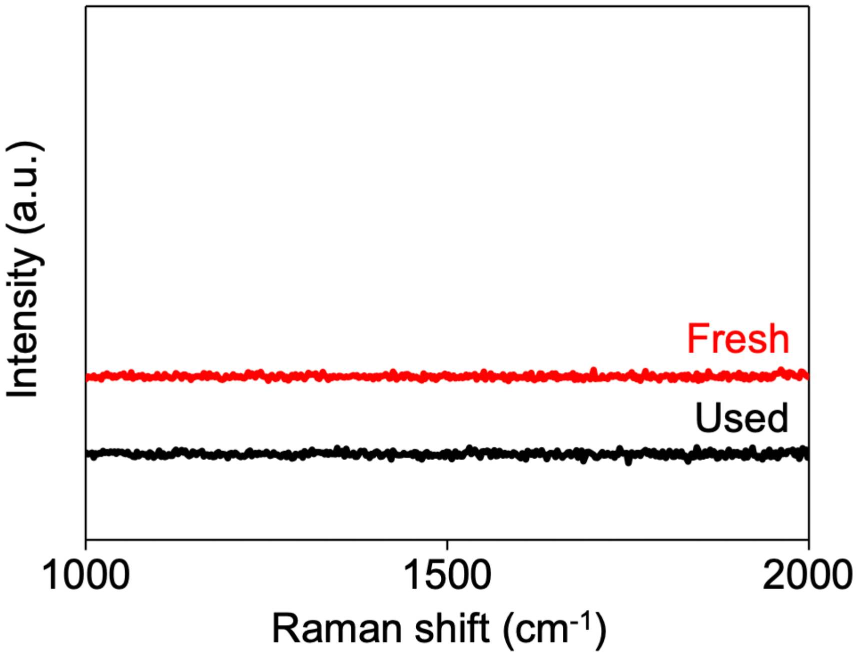

Figure S6. Representative Raman spectra of Pt_1/GaN before and after reaction.

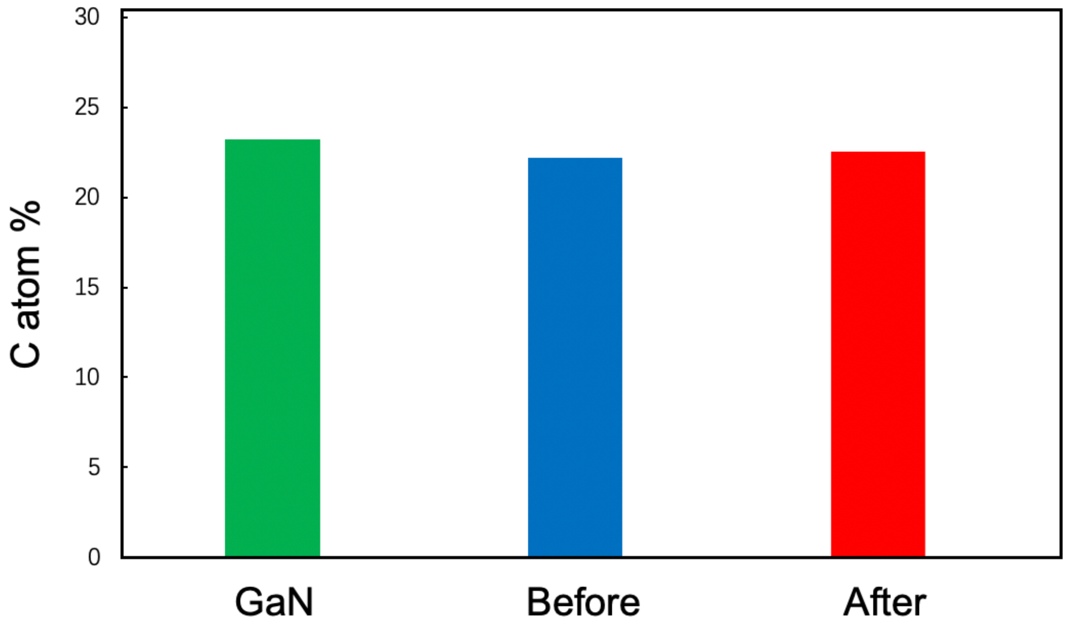

Figure S7. Carbon element content on the catalyst surface of GaN and Pt_1/GaN before and after reaction.

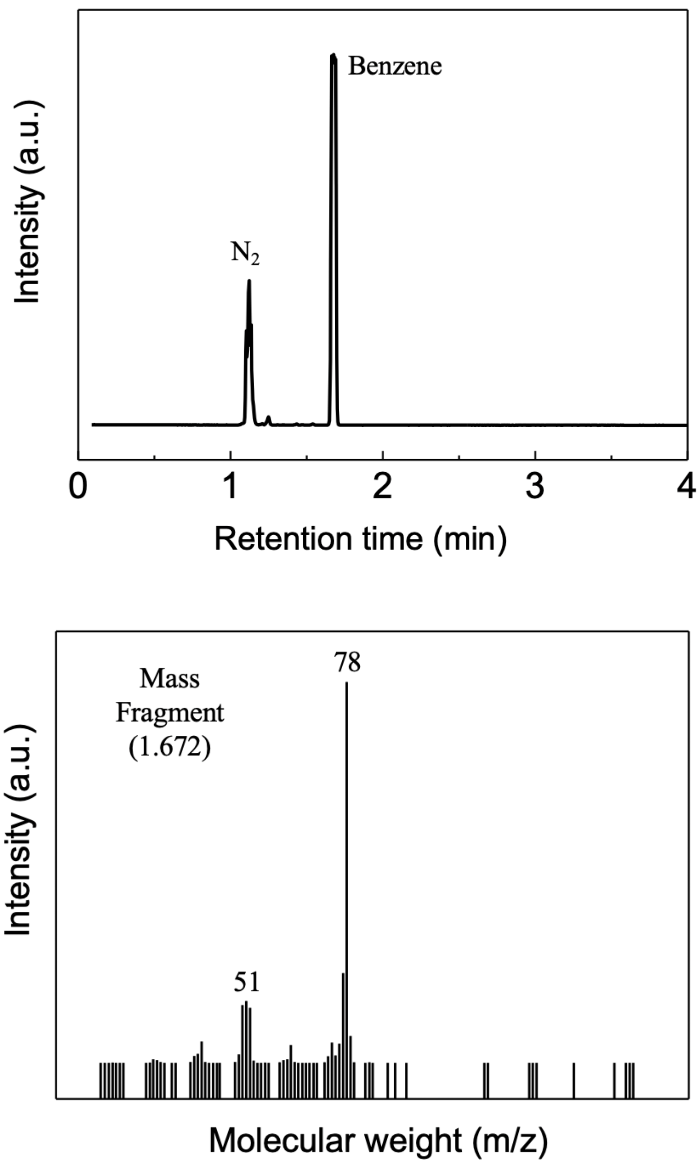

Figure S8. Representative GC-MS chromatography result for the gas sample after the hydrogenation of benzene over Pt_1/GaN at 300 °C. It is noted that rather low concentration of H_2 molecular is hard to trigger benzene hydrogenation, leading to zero cyclohexane production. Reaction conditions: 20 mg of catalyst, 4.5 μmol of benzene, 27 μmol of H_2, 2 h.

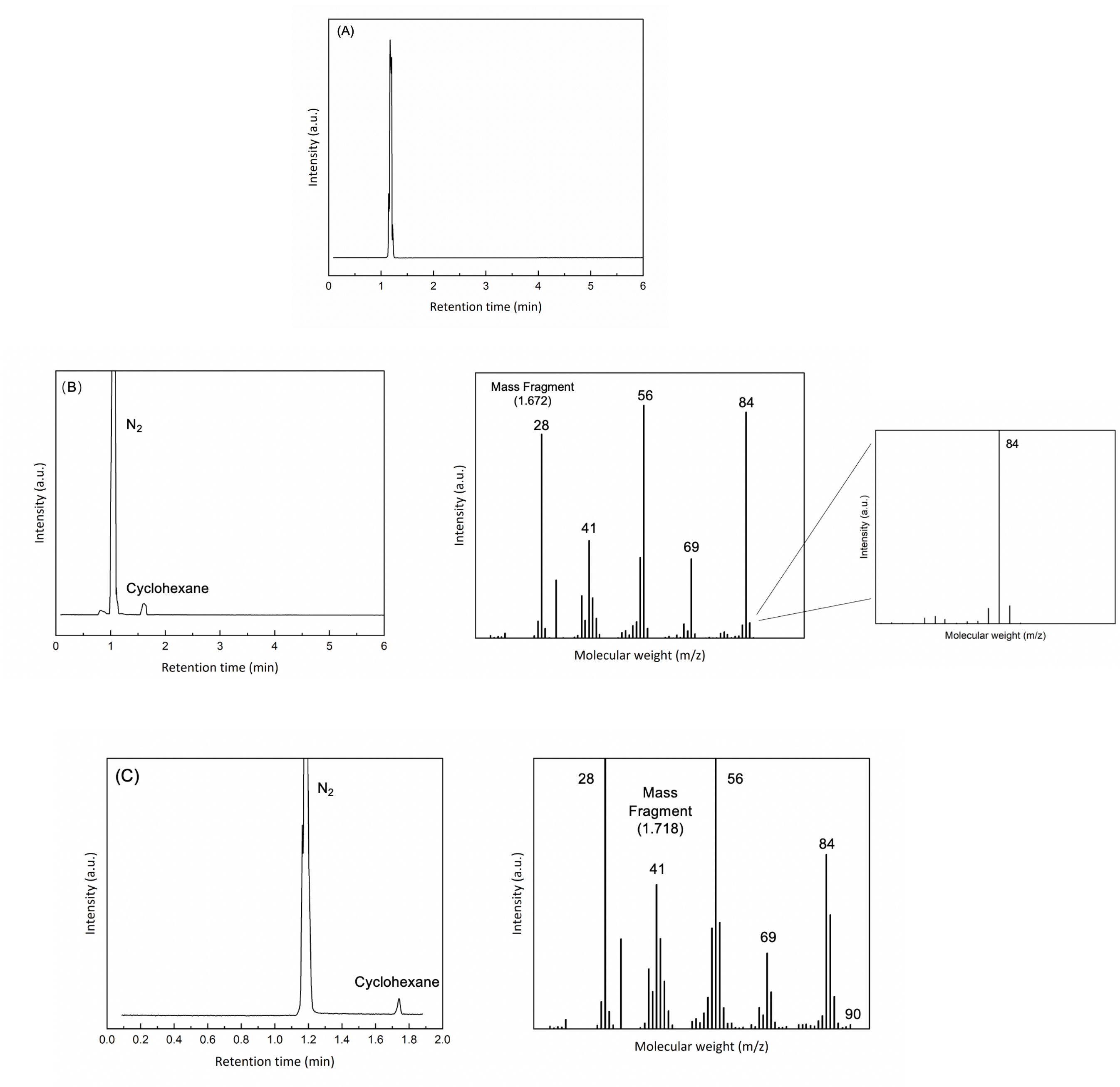

Figure S9. Representative GC-MS chromatography result for the gas sample over Pt_1/GaN at 300 °C. Reaction conditions: 20 mg of catalyst, 1 atm, 50 mL of reagent gas, 2 h. Reagent gas: (**A**) Ar gas, (**B**) ^{12}C methane, (**C**) mixture of ^{12}C and ^{13}C methane (50/50).

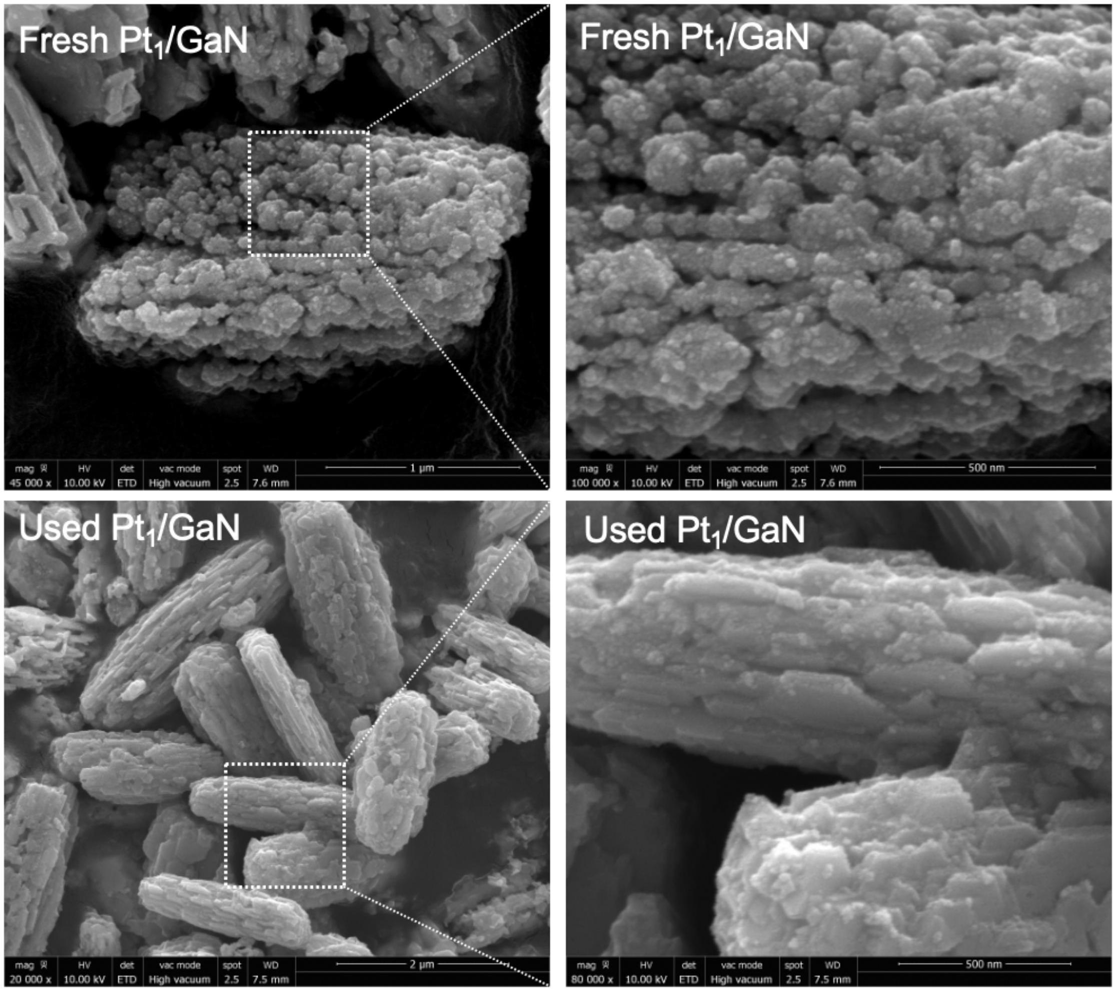

Figure S10. Typical SEM images of Pt_1/GaN before and after reaction.

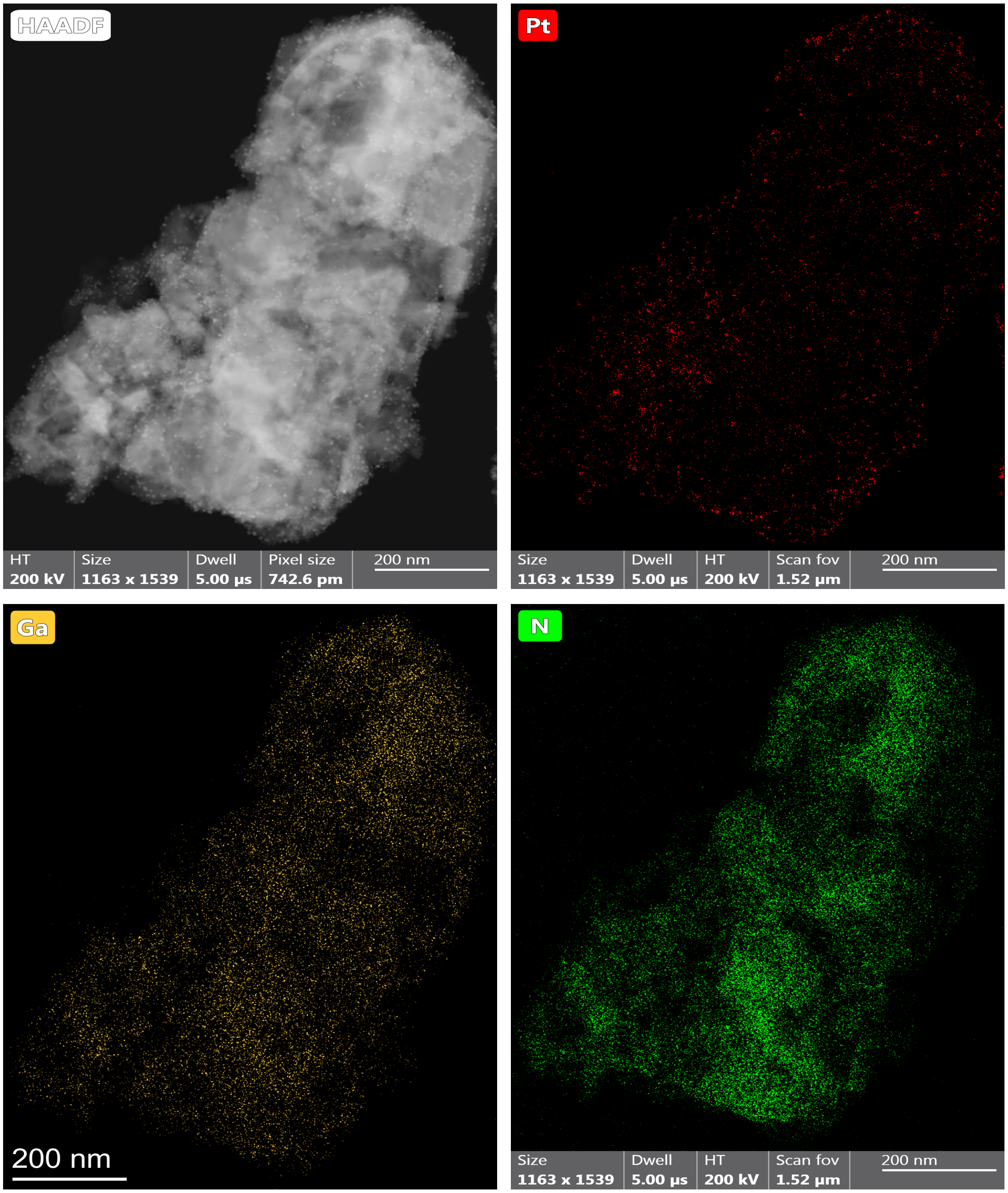

Figure S11. Typical HAADF-STEM images and EDX mapping of Pt_1/GaN after reaction.

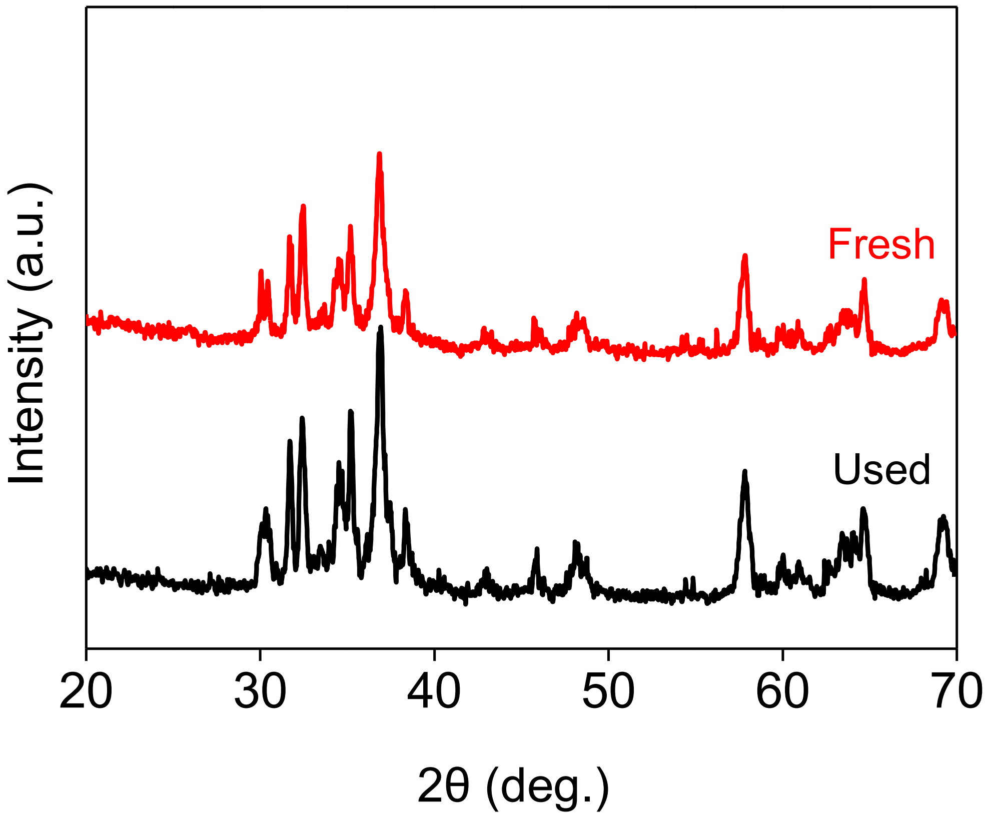

Figure S12. Representative XRD spectroscopy of Pt_1/GaN before and after reaction.

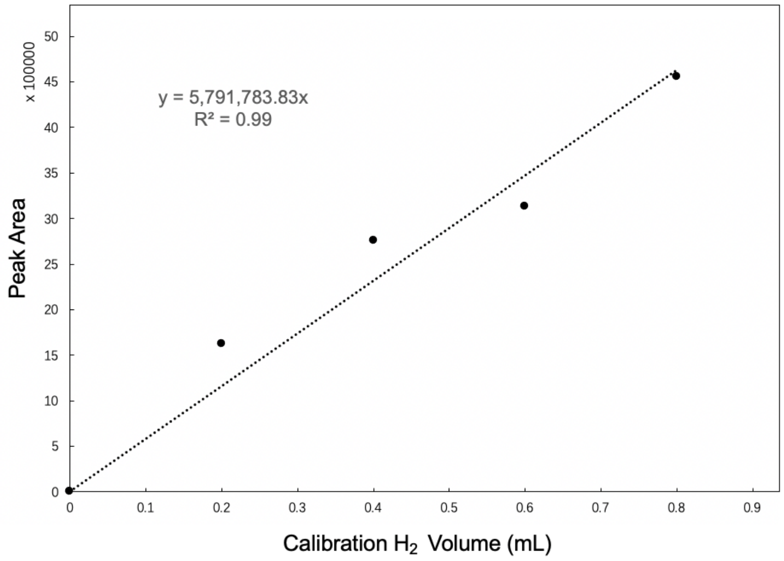

Figure S13. Calibration Curve obtained for hydrogen gas quantification.

Table S1. Results of various catalysts for the methane to cyclohexane conversion. [a]

Entry	Catalyst	Temperature (°C)	Selectivity of cyclohexane (%)	Yield (μmol/g)			
				Cyclohexane	Benzene	Toluene	Xylene
1	GaN	300	/	n.d.[b]	0.094	traces	traces
2	TiO_2	300	/	n.d.	n.d.	n.d.	n.d.
3	Ga_2O_3	300	/	n.d.	0.012	n.d.	n.d.
4	ZnO	300	/	n.d.	0.045	0.0085	n.d.
5	Pt_1/TiO_2	300	/	n.d.	n.d.	n.d.	n.d.
6	Pt_1/Ga_2O_3	300	47.83	0.11	0.12	n.d.	n.d.
7	Pt_1/ZnO	300	42.31	0.11	0.15	n.d.	n.d.
8	Pt_1/C_3N_4	300	/	n.d.	0.08	n.d.	n.d.
9	Pt_1/GaN	300	90.14	6.49	0.71	n.d.	n.d.
10	$Pt_{0.5}/GaN$	300	/	n.d.	0.83	n.d.	n.d.
11	Pt_2/GaN	300	61.42	2.69	0.68	1.01	n.d.
12	Pt_4/GaN	300	42.31	0.11	0.15	n.d.	n.d.
13	PtO/GaN	300	/	n.d.	0.21	n.d.	n.d.
14	Pt_1/GaN	250	97.22	0.084	0.0024	n.d.	n.d.
15	Pt_1/GaN	350	37.05	3.32	5.64	n.d.	n.d.
16	Pt_1/GaN	400	12.72	2.39	4.22	4.85	7.44
17[c]	Pt_1/GaN	300	93.52	11.55	0.80	n.d.	n.d.
18[d]	Pt_1/GaN	300	92.23	41.42	3.49	n.d.	n.d.

[a] Reaction conditions were as follows: 20 mg of catalyst, 2 mmol of methane, 2 hours. After the reaction, the reaction products were analyzed and quantified by GC-MS with FID detector.

[b] Not detectable.

[c] The catalyst activity test was performed in the 100 mL reactor.

[d] The catalyst activity test was performed in the 500 mL reactor.

Table S2. Results of methane transformation into cyclohexane over Pt_1/GaN.[a]

Entry	Catalyst	Time (min)	Selectivity of cyclohexane (%)	Yield (μmol/g)			
				Cyclohexane	Benzene	Toluene	Xylene
1	Pt_1/GaN	10	/	n.d.[b]	1.43	n.d.	n.d.
2	Pt_1/GaN	20	59.62	2.54	1.72	n.d.	n.d.
3	Pt_1/GaN	30	89.51	3.67	0.43	n.d.	n.d.
4	Pt_1/GaN	60	87.66	8.81	1.24	n.d.	n.d.
5	Pt_1/GaN	90	86.58	8.00	1.24	n.d.	n.d.
6	Pt_1/GaN	120	86.26	9.79	1.56	n.d.	n.d.
7	Pt_1/GaN	150	83.67	7.48	1.46	n.d.	n.d.
8	Pt_1/GaN	180	87.31	9.56	1.39	n.d.	n.d.
9	Pt_1/GaN	300	90.73	28.78	2.94	n.d.	n.d.

[a] Reaction conditions were as follows: 20 mg of catalyst, 2 mmol of methane, 300 ^{o}C; After the reaction, the organic products were analyzed and determined by chromatography GC-MS with FID detector.

[b] Not detectable.

Table S3. Results of methane transformation into cyclohexane over the mixture of commercial Pt/C and GaN.[a]

Entry	Catalyst	Time (min)	Selectivity of cyclohexane (%)	Yield (μmol/g)			
				Cyclohexane	Benzene	Toluene	Xylene
1	5%Pt/C+GaN	30	/	n.d.[b]	0.0075	n.d.	n.d.
2	5%Pt/C+GaN	60	/	n.d.	0.063	n.d.	n.d.
3	5%Pt/C+GaN	90	/	n.d.	0.083	n.d.	n.d.
4	5%Pt/C+GaN	120	/	n.d.	0.10	n.d.	n.d.
5	5%Pt/C+GaN	150	/	n.d.	0.15	n.d.	n.d.
6	5%Pt/C+GaN	180	/	n.d.	0.17	n.d.	n.d.
7	5%Pt/C+GaN	300	/	n.d.	0.18	n.d.	n.d.

[a] Reaction conditions were as follows: 20 mg of catalyst, 2 mmol of methane, 300 ^{o}C; After the reaction, the organic products were analyzed and determined by chromatography- GC-MS with FID detector.
[b] Not detectable.

Table S4. Reusability results for methane transformation over Pt_1/GaN.[a]

Entry	Catalyst	Selectivity of cyclohexane (%)	Yield (μmol/g)			
			Cyclohexane	Benzene	Toluene	Xylene
1	Pt_1/GaN	88.67	6.34	0.81	n.d.[b]	n.d.
2	Pt_1/GaN	86.13	6.52	1.05	n.d.	n.d.
3	Pt_1/GaN	88.00	6.53	0.89	n.d.	n.d.
4	Pt_1/GaN	87.02	6.30	0.94	n.d.	n.d.
5	Pt_1/GaN	87.41	6.39	0.92	n.d.	n.d.

[a] Reaction conditions were as follows: 20 mg of catalyst, 2 mmol of methane, 300 °C; After the reaction, the organic products were analyzed and determined by chromatography- GC-MS with FID detector.

[b] Not detectable.

Table S5. Comparison of catalytic performance of different catalysts for direct methane to cyclohexane conversion.

Entry	Catalysts	Reaction Condition	Selectivity to cyclohexane (%)	Cyclohexane productivity (μmol/g)	Ref.
1	Pt_1/GaN	300 ºC, 1 atm	90.14	6.49	This work
2	GaN[2]	650-710 ºC flow (GHVS of 114-720 $ml_N\ g_{cat}^{-1}h^{-1}$), 1atm	Detectable	/	*Catal. Commun.* **2018**, *106*, 16-19.
3	GaN[3]	700 ºC, 1atm	1.1	0.275	*Angew. Chem.,* **2014**, *126*, 14330-14333.

References

1. Fang, S. *et al.* Tuning the Charge Transfer Dynamics of the Nanostructured GaN Photoelectrodes for Efficient Photoelectrochemical Detection in the Ultraviolet Band. *Advanced Functional Materials* **31**, 2103007 (2021).

2. Dutta, K., Li, L., Gupta, P., Gutierrez, D. P., Li, C.-J. & Kopyscinski, J. Direct non-oxidative methane aromatization over gallium nitride catalyst in a continuous flow reactor. *Catalysis Communications* **106**, 16–19 (2018) (Corrigendum: Dutta, K.; Li, L.; Guptaa, P.; Gutierreza, D. P.; Li, C.-J.; Kopyscinski, J. *Catal. Commun*., **2018**, *111*, 108).

3. Li, L., Mu, X., Liu, W., Kong, X., Fan, S., Mi, Z. & Li, C.-J. Thermal non-oxidative aromatization of light alkanes catalyzed by gallium nitride. *Angew. Chem. Int. Ed.* **126**, 14330-14333 (2014).
